# Influenza-Associated Disease Burden in Kenya: A Systematic Review of Literature

**DOI:** 10.1371/journal.pone.0138708

**Published:** 2015-09-23

**Authors:** Gideon O. Emukule, John Paget, Koos van der Velden, Joshua A. Mott

**Affiliations:** 1 Centers for Disease Control and Prevention, Kenya Country Office, Nairobi, Kenya; 2 Netherlands Institute for Health Services Research, NIVEL, Utrecht, The Netherlands; 3 Radboud University Medical Center, Department of Primary and Community Care, Nijmegen, The Netherlands; 4 Influenza Division, National Center for Immunization and Respiratory Diseases, US Centers for Disease Control and Prevention, Atlanta, GA, United States of America; 5 US Public Health Service, Rockville, Maryland, United States of America; New York City Department of Health and Mental Hygiene, UNITED STATES

## Abstract

**Background:**

In Kenya data on the burden of influenza disease are needed to inform influenza control policies.

**Methods:**

We conducted a systematic review of published data describing the influenza disease burden in Kenya using surveillance data collected until December 2013. We included studies with laboratory confirmation of influenza, well-defined catchment populations, case definitions used to sample patients for testing and a description of the laboratory methods used for influenza testing. Studies with or without any adjustments on the incidence rates were included.

**Results:**

Ten studies reporting the incidence of medically-attended and non-medically attended influenza were reviewed. For all age groups, the influenza positive proportion ranged from 5–10% among hospitalized patients, and 5–27% among all medically-attended patients (a combination of in- and outpatients). The adjusted incidence rate of hospitalizations with influenza among children <5 years ranged from 2.7–4.7 per 1,000 [5.7 per 1,000 in children <6 months old], and were 7–10 times higher compared to persons aged ≥5 years. The adjusted incidence of all medically-attended influenza among children aged <5 years ranged from 13.0–58.0 per 1,000 compared to 4.3–26.0 per 1,000 among persons aged ≥5 years.

**Conclusions:**

Our review shows an expanding set of literature on disease burden associated with influenza in Kenya, with a substantial burden in children under five years of age. Hospitalizations with influenza in these children were 2–3 times higher than reported in the United States. These findings highlight the possible value of an influenza vaccination program in Kenya, with children <5 years and pregnant women being potentially important targets.

## Introduction

Human influenza infections are a major cause of morbidity and mortality worldwide [1–3]. Although risk factor data from tropical climates are limited, young children (<5 years), pregnant women, the elderly, and persons with underlying medical conditions have been shown to be at increased risk of severe disease [1, 4]. A recent study estimated that there were 20 million cases of influenza associated with pneumonia; 1 million cases of influenza associated with severe pneumonia; and 28000–111500 deaths associated with influenza among children aged <5 years globally in 2008, with 99% of these deaths occurring in the developing world [5]. A summary of existing direct estimates of influenza disease burden in tropical and developing countries is needed to validate global modeling efforts that suggest a disproportionate burden in these countries.

In Kenya, influenza surveillance was established partly in response to the global emerging threat of avian influenza A(H5N1)[6, 7]. As is the case with other tropical and sub-tropical countries, influenza viruses circulate in Kenya for most of the year [7–9] and morbidity (hospitalization and outpatient) burden of influenza have only recently been described [10–18].

An improved understanding of disease burden in Kenya relating to morbidity, mortality, and economic losses is needed to support decisions involving the allocation of limited resources toward influenza control programs. The Kenyan Ministry of Health (KMoH) has released its first ever influenza vaccination policy [19] and this has necessitated the publication of an overview of the burden of influenza in Kenya to inform initial vaccination pilot activities.

In this article, we review existing data on the influenza disease burden in Kenya using data collected until December 2013. We summarize published data describing the health burden of human influenza collected through population-based influenza surveillance systems in Kenya. We also discuss the various disease burden estimation methods used and provide suggestions for future research strategies that will help to generate additional data needed to inform influenza control strategies.

## Methods

### Search strategy and selection criteria

Our objective is to provide a comprehensive overview of the disease burden of influenza in Kenya. We carried out a literature review with specific search terms; "Kenya" and each of the following words "Influenza", "Respiratory", "Pneumonia", "Severe Acute Respiratory Illness", and "Influenza-like Illness". We searched PubMed and EMBASE (Ovid) for studies–with no language restrictions—that contained original data and were conducted until December 2013. The search was last conducted on March 23, 2015. We created a master list of the search results from these two search databases with two variables; author names and study title. We then removed duplicates. Titles from these search results were reviewed for the presence of any of the following key words; “Kenya”, “Influenza”, “respiratory”, “pneumonia”, “influenza-like illness”, “acute lower respiratory infection”, “acute upper respiratory infection”, “mortality”, “deaths”, “hospitalization”, “hospital admission”, and “outpatient”. Abstracts of articles that contained at least one of these search words were then reviewed by one researcher (GOE) and included if they contained information on disease burden of influenza in Kenya.

Only studies with original data collected before December 2013 were included. We considered studies for inclusion: (i) if they reported incidence rates of hospitalization and/or outpatient visits associated with influenza-like illness (ILI), acute respiratory illness (ARI), acute lower respiratory illness (ALRI), severe respiratory illness (SARI), and severe or very severe pneumonia using laboratory confirmed influenza cases; (ii) if they had well-defined catchment populations or estimations of the population-at-risk [using any of population-based disease surveillance systems, health demographic surveillance systems (HDSS), national population census data, or population registration records]; (iii) if they provided the case definition used to sample patients for testing; and (iv) if they provided a description of the laboratory testing methods used. Studies that presented adjusted or unadjusted (crude) incidence rates were included in the review (see S1 File, for a summary of the formulae used for the adjustments). All reported rate adjustments are indicated for each study. Additionally, we scanned the reference lists and titles of articles selected for review using the criteria defined above.

**Fig 1:**
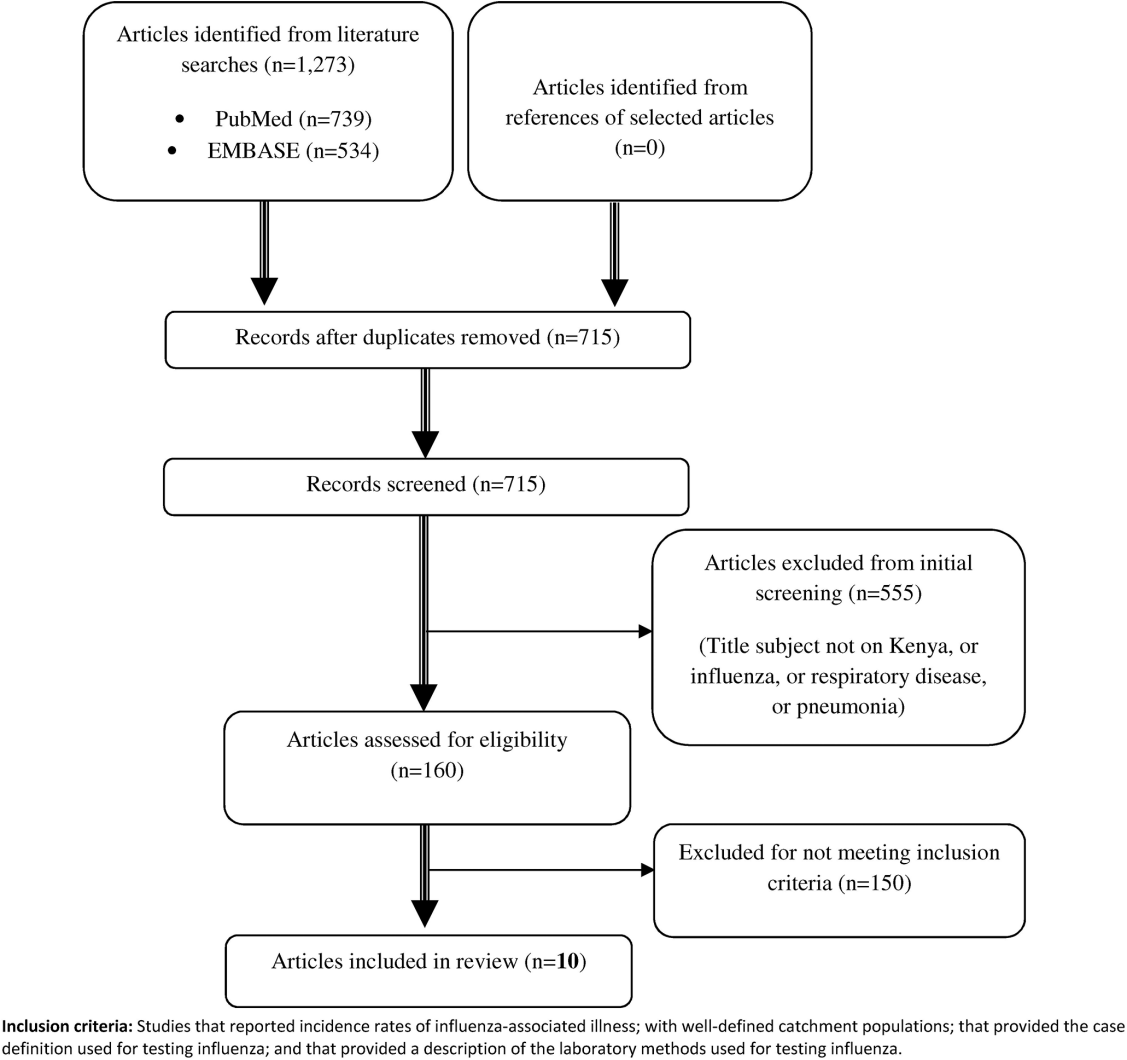
Overview of the different phases of literature selection.

A flow chart with details of the process followed in selecting the articles that were reviewed, and the number of articles included–and excluded from this review is provided in Fig 1.

### Data extraction, analysis and reporting

Data extraction was performed by one researcher (GOE) using a template that collected details on study characteristics [title, author(s), publication year, place of study, study participants age group, syndromes used for case identification, and adjustment factors used for calculating incidence rates]. The adjustments applied to the rates were limited to one or more of the following: (i) patients who met the swabbing criteria but were not tested for influenza; (ii) cases in the community who met the specific case definition but did not seek healthcare at the study hospital/clinic; and (iii) asymptomatic detection of influenza using controls to determine illness attributable to influenza. In this review, we reported both adjusted and unadjusted influenza burden disease rates, but our primary measure was the adjusted rates as these were more accurate estimates of the disease burden.

The outcome measures that we considered were incidence of: (i) hospitalizations with influenza; (ii) medically-attended influenza (outpatient and inpatient combined); and (iii) non-medically attended influenza. We also, as a secondary measure, reported on the proportion of those who tested positive for influenza if data on incidence of influenza illness were reported. Other than the conditions defined in the inclusion criteria, no further quality assessments were applied to the reviewed articles. All incidence rates were reported per 1,000 persons or person-years. Adjusted rates reported in our paper included at least an adjustment for patients who met the swabbing criteria but were not tested, which was the most commonly applied adjustment in the studies that we reviewed.

Data from the articles reviewed were summarized as ranges (minimum—maximum), where two or more studies were involved, and presented in tables by the following domains: (i) proportions testing positive for influenza; (ii) hospitalizations with influenza; (iii) medically-attended influenza; and (iv) non-medically attended influenza.

## Results

### Search results and description of methods used

There were a total of 1,273 search records returned, including duplicates (PubMed = 739; and EMBASE = 534) (Fig 1). After removing duplicates, there were 715 unique articles that were returned from the search and among these were 555 (78%) were on subjects not related to influenza or respiratory illness and were excluded in the first round of screening. Of the remaining 160 articles, there were 51 articles on an influenza related subject; 67 on pneumonia, and 42 on a broader respiratory subject other than influenza and pneumonia. Of these 160 articles, 10 met the inclusion criteria for this review.

**Fig 2:**
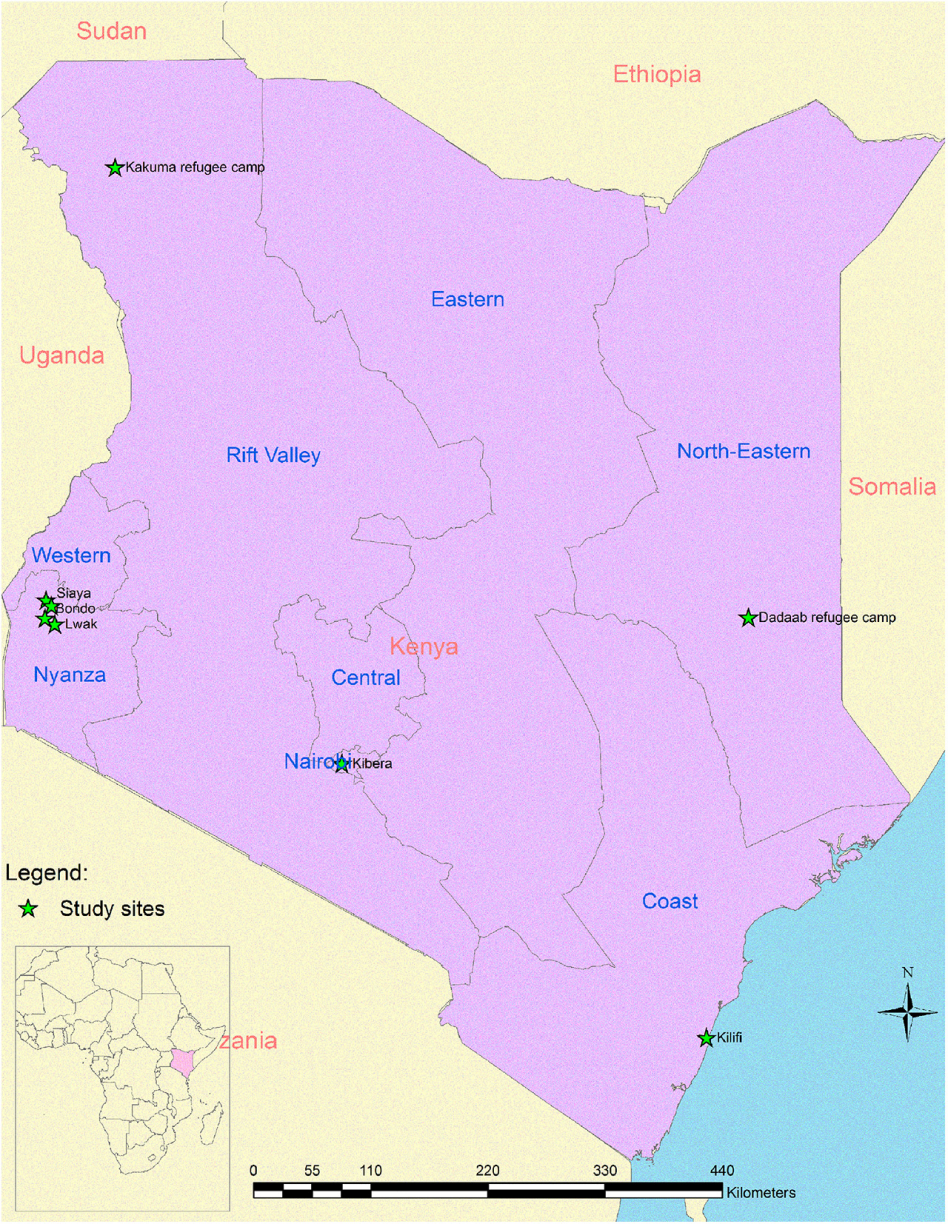
Map of Kenya showing the study sites which generated data that was used in the reviewed papers.

Fig 2 shows the location of the study sites that generated the data that were used in the analysis for articles that we reviewed. Eight of the 10 articles reviewed were based on surveillance data collected by the Kenya Medical Research Institute (KEMRI) and the Centers for Disease Control and Prevention (CDC) [10–14, 17, 18, 20]. The remaining two articles were based on data collected by the KEMRI and Wellcome Trust Research Program [15, 16]. Of the ten articles reviewed, one was published in 2010 [15], five in 2012 [11–13, 16, 17], two in 2013 [10, 14], one in 2014 [18] and one in 2015 [20]. Case definitions used in two of these articles included data on severe or very severe pneumonia [15, 16]; six included data on severe acute respiratory illness (SARI) or acute lower respiratory illness (ALRI)[10, 11, 14, 17, 18, 20]; two included data on influenza-like illness (ILI) [11, 18]; and two included data on acute respiratory illness (ARI) [12, 13].

The case definitions for the respiratory syndromes used in identifying the cases to be tested for influenza varied (Table A in S1 Table). Six of the articles reviewed used mid-year population data from well-defined catchment areas as denominators, either from a HDSS, refugee camp records, or national census data for estimation of incidence while the rest used person-time years of follow-up calculated from a population-based surveillance system. All ten articles that reported influenza A and/or B testing, used reverse transcriptase polymerase chain reaction (RT-PCR) testing methods. The data reported in all the papers reviewed were collected year-round, and all (except one [15]) of the papers had multiple years of data included. All–except one [15]—of the articles included data collected during the 2009 pandemic period.

Most (9/10) of the articles reported data on the proportions of patients who tested positive for influenza (Table B in S1 Table). Six of the nine articles reported rates of hospitalization with influenza (Table 1), four reported rates of medically-attended influenza (both in- and outpatients) (Table 2), and two reported incidence of non-medically attended SARI or ILI [10, 18] (Table 3). None of the studies reviewed had data on influenza mortality in Kenya.

**Table 1:** Average annual incidence rates of hospitalization with influenza for different respiratory syndromes (per 1,000 persons or person-years) in Kenya.

Author(s)	Syndrome type	Adjustment used	Study site	Age group	Incidence^a^ Range^b^
Berkley et al. (2010)[15] and Onyango et al. (2012)[16]	Hospitalized Severe or very severe pneumonia	None stated	Kilifi	< 1 yr	1.5–2.4
				<5 yrs	0.6–0.8
Ahmed et al. (2012)[17]	Hospitalized SARI	None stated	Kakuma & Dadaab refugee camp	< 1 yr	10.3–12.3
				< 5 yrs	4.2–5.6
Fuller et al. (2013)[10] and Emukule et al. (2014)[18]	Hospitalized SARI	Healthcare seeking; those with syndrome who did not have swabs tested for influenza virus	Siaya, Western Kenya	<6 mos	5.7
				<5 yrs	2.7–4.7
				≥5 yrs	0.2–0.4
				All ages	0.7–1.1
Feikin et al. (2012)[12]	Hospitalized ARI	Rates adjusted for those hospitalized with ARI who did not have swabs tested for influenza	Bondo, Western Kenya	<1 yr	1.4
				<5 yrs	1.4
				All ages	0.6
All studies	All syndromes	With or without any adjustment	All study sites	<6 mos	5.7
				<1 yr	1.4–12.3
				<5 yrs	0.6–5.6
				≥5 yrs	0.2–0.4
				All ages	0.6–1.1

Abbreviations

SARI = Severe acute respiratory illness

ARI = Acute respiratory illness.

^a^Incidence reported per 1,000 persons or person-years

^b^Range is the minimum-maximum in cases where two or more studies were involved.

**Table 2:** Average annual incidence rates of medically-attended influenza A and/or B (hospitalized and outpatient) per 1,000 persons or person-years in Kenya.

Author(s)	Syndrome type	Adjustment used	Study site	Age group	Incidence^a^ Range^b^
Katz et al. (2012)[11]	In- and outpatient ALRI	Adjusted for those with ALRI who were not tested for influenza	Kibera and Lwak	< 1 yr	32.8–42.1
				<5 yrs	22.0–40.5
				≥5 yrs	12.0–15.8
				All ages	13.7–23.0
Feikin et al. (2013)[14]	In- and outpatient SARI	Adjusted for healthcare seeking by extrapolating from those with ARI^ǂ^ at household visit who sought care at a clinic besides the study clinic and for the pathogen-attributable fraction (PAF^¥^)	Lwak, Western Kenya	<5 yrs	58.0
Breiman et al. (2015)[20]	Outpatient SARI	Adjusted for healthcare seeking for SARI at the study clinic and for the pathogen-attributable fraction (PAF^¥^).	Kibera	<5 yrs	13.0
Feikin et al. (2012)[13]	In- and outpatient ARI	Adjusted for healthcare seeking by extrapolating from those with ARI^ǂ^ at household visit who sought care at a clinic besides the study clinic and for the pathogen-attributable fraction (PAF^¥^)	Lwak, Western Kenya	≥5 yrs	26.0
Emukule et al. (2014)[18]	Outpatient ILI	Adjusted for those with ILI who were not tested for influenza	Ting'wang'i, Western Kenya	<6 mos	16.2
				<5 yrs	21.8
				≥5 yrs	4.3
				All ages	7.2
All studies	All syndromes	With any adjustment	All study sites	<6 mos	16.2
				< 1 yr	32.8–42.1
				<5 yrs	21.8–58.0
				≥5 yrs	4.3–26.0
				All ages	7.2–23.0

Abbreviations

SARI = Severe acute respiratory illness

ALRI = Acute lower respiratory illness

ILI = influenza-like illness

ARI = Acute respiratory illness

^a^Incidence reported per 1,000 persons or person-years

^b^Range is the minimum-maximum in cases where two or more studies were involved

^ǂ^ARI in home was defined as cough, difficulty breathing or chest pain and reported fever

^¥^Adjusted rates downward for asymptomatic detection of influenza in controls.

**Table 3:** Non-medically attended average annual incidence rates of Influenza reported for different respiratory syndromes (per 1,000 persons or person-years) in Kenya.

Author(s)	Syndrome type	Adjustment used	Study site	Age group	Incidence^a^ Range^b^
Fuller et al. (2013)[10]; Emukule et al. (2014)[18]	Non-medically attended SARI	Adjusted for persons with pneumonia who did not seek care from health utilization survey (HUS)	Siaya	<6 mos	6.2
				<5 yrs	2.9–5.1
				≥5 yrs	0.4–0.8
				All ages	0.9–1.4
Emukule et al. (2014)[18]	Non-medically attended ILI	Adjusted for persons with ARI who did not seek care from HUS	Ting'wang'i	<6 mos	22.3
				<5 yrs	30.1
				≥5 yrs	5.4
				All ages	9.1

^a^Incidence reported per 1,000 persons or person-years

^b^Range is the minimum-maximum in cases where two or more studies were involved.

### Proportions testing positive for influenza

The proportions of those who tested positive for influenza A and/or B varied among the studies included (Table B in S1 Table). These ranged from 4.9% to 26.7% for all medically-attended patients [4.9% to 13.7% among children <5 years; 14.0% to 20.5% among persons ≥5 years; and 9.8% to 26.7% in studies that reported proportions among patients of all ages]. Among hospitalized patients who were tested, the proportion of those who tested positive for influenza A and/or B ranged from 5% to 10%.

### Incidence rate of hospitalization with influenza

Incidence rates of hospitalization with influenza varied among the studies, which were implemented during different years, and used varying case definitions, and adjustments factors (Table 1 and Table C in S1 Table). There were six studies that reported incidence rates of hospitalization with influenza. Two studies conducted among children with severe or very severe pneumonia reported similar incidence rates among children <5 years [0.8 cases per 1,000 in the first study, and 0.6 cases per 1,000 (95% CI 0.5–0.7) in the second study] [15, 16]. No adjustments for people with pneumonia who were not tested were reported in these two studies. Unadjusted rates of influenza among hospitalized children in the age group <5 years were also reported in a study conducted in two refugee sites. These unadjusted rates ranged 4.2–5.6 cases per 1,000 for influenza A viruses and 1.1–1.4 for influenza B viruses.

Adjusted incidence rates of hospitalization with influenza among children of the age group of <5 years who presented with SARI ranged from 2.7–4.7 per 1,000 [10, 18]. Adjusted incidence rates of hospitalization with influenza among persons aged ≥5 years who presented with SARI ranged from 0.2–0.4 per 1,000 among persons aged ≥5 years, and were lower compared to those of children in the age group of <5 years. In Western Kenya there was a high incidence rate of hospitalization with influenza among children <6 months [5.7(95% CI 2.4–13.8) per 1,000][18].

### Incidence rate of medically-attended influenza

Over the study period covered in our review, there were three publications that reported broader medically-attended (combining in- and outpatients) influenza incidence rates. Two of the publications were based on medically-attended ALRI [11, 14]; and another on medically-attended ARI [13]. Two studies reported medically-attended influenza incidence rates only for outpatients [18, 20]. The adjusted incidence rates of medically-attended influenza ranged from 21.8–58.0 per 1,000 child-years for children in the age group of <5 and 4.3–26.0 for persons aged ≥5 years (Table 2 and Table D in S1 Table).

A study that was conducted in a peri-urban informal settlement in Nairobi (Kibera) and a rural site in Western Kenya (Lwak) among patients who sought care for ALRI as inpatients and/or outpatients, reported higher adjusted incidence rates for influenza among children <5 years in the rural site [40.5 (95% CI 31.2–52.6)] compared to the urban site [22.0 (95% CI 17.7–26.6)]. However, similar results were reported among persons ≥5 years [15.8 (95% CI 14.1–17.7) vs. 12.0 (95% CI 10.3–13.3)] in the rural and urban sites respectively [11] (Table D in S1 Table).

### Incidence rate of non-medically attended influenza

Only two of the studies reviewed (both conducted in Western Kenya) estimated non-medically attended incidence rates of influenza (two reported non-medically attended SARI in Siaya [10, 18]; and one reported non-medically attended ILI [18]). The incidence of influenza with non-medically attended severe ARI ranged from 2.9–5.1 per 1,000 among children <5 years, and 0.4–0.8 among persons aged ≥5 years. In the one study that estimated incidence of influenza among non-medically attended ILI cases, there were an estimated 30.1 cases of influenza per 1,000 (95% CI 27.3–33.3) among children <5 years and 5.4 cases per 1,000 (95% CI 4.9–6.0) among persons aged ≥5 years [18] (Table 3 and Table E in S1 Table).

## Discussion

We have provided a comprehensive summary of available data on disease burden of influenza in Kenya and we show that influenza is an important cause of respiratory infection-associated morbidity, especially among younger children under the age of five years. Indeed, both adjusted and unadjusted incidence rates of hospitalization with influenza [10, 17, 18], and outpatient visits[18] were higher than those that have been reported in United States and European countries during similar time periods [21–32].

We also note that the published literature on the burden of influenza in Kenya is limited but expanding. Eight of the ten papers that we reviewed were published within the last three years (2012–2014)–including two studies that published data on the post- pandemic A(H1N1) period. This could be attributed to the interest generated by the threat of avian and pandemic influenza.

Our review showed that there were an estimated 2.7–4.7 cases of influenza per 1,000 among children <5 years who were hospitalized with severe acute respiratory illness (SARI). These were 7–10 times higher compared to those in persons aged ≥5 years. A study that estimated disease burden among hospitalized children <6 months in Western Kenya reported that there were 5.7 cases of influenza per 1,000 [18]. This is consistent with data from several other countries [22, 23, 33], and shows a considerable burden of disease in young infants for whom influenza vaccination is not recommended, and also highlights the rationale for targeting pregnant mothers for influenza vaccination [34]. Whereas pregnant mothers have been shown to be at increased risk of complications associated with influenza [35], vaccinating them may not only be beneficial to them but could also offer protection to their young infants—for whom no influenza vaccine is currently licensed—through breastfeeding and trans-placental antibody transfer [36, 37].

The incidence rates of hospitalization with influenza among children <5 years reported at the two refugee sites (Kakuma and Dadaab)–without adjustments for eligible cases who were not tested—were higher than rates reported elsewhere in Kenya. This could be due to the unique challenges experienced by the populations in refugee settings, such as population density, which could make them more vulnerable to exposure to respiratory infections [38, 39]. While the incidence rates of hospitalization with influenza are similar to those reported in South Africa [40], Asia [41–46], Latin America [47], some of the reported rates were up to seven times higher than rates reported in the United States [21–26] and Europe [27, 28]. The incidence rates of hospitalization with influenza children <6 months in Kenya, for example, were 2–3 times higher than rates reported in the United States [23].

The adjusted incidence of medically-attended (outpatient + inpatient) influenza among children <5 years ranged from 21.8 to 58.0, and 4.3 to 26.0 per 1,000 among persons aged ≥5 years in different studies. These rates were similar to rates reported in Asia [48], but up to 2–4 times higher than annual estimates reported in Europe [29–31], and up to 2–8 times higher than rates reported in the United States [32].

The incidence of non-medically attended severe ARI associated with influenza suggested a burden of disease that was similar to the medically-attended burden. As reported in the health utilization survey conducted in Western Kenya, 52% of children <5 years and 66% of persons ≥5 years who reported to have had pneumonia did not seek care at a hospital [49]. The similarity between the medically-attended and non-medically attended incidence not only underscores the fact that there is a considerable burden of non-medically attended influenza, but also highlights the low levels of health-care seeking for respiratory illness in Kenya [49]. These findings also suggest that surveillance limited to the health care setting will not capture the entire burden of influenza severe respiratory illness in contexts such as Kenya.

The studies reviewed included various adjustments for patients who met the case definitions but were not tested for influenza [10–12, 18]; for those who sought health-care at a facility other than the one used for estimating the incidence rates [13, 14]; and for asymptomatic detection of influenza among controls [13, 14]. The first two adjustments would serve to increase the crude incidence to account for persons who met the case definition and were not tested for influenza or those who did not seek care; while the latter would drive the rate downwards by only accounting for the cases for which the virus was the likely cause disease. Other than the case definition for ILI developed by the World Health Organization (WHO) which was more commonly applied across the different studies [50], the case definitions used for SARI, ALRI and ARI also substantially varied across the studies reviewed. In order to facilitate disease burden comparisons over time, it would have been helpful if researchers also presented their data using standard case definitions as recommended by WHO[51]; and unadjusted rates in addition to those where adjustments were applied. These standardizations, in addition to presenting data in age groups that may be aggregated to WHO recommended age categories [<2, 2–4, 5–14, 15–49, 50–64, and ≥65 years] [51] would help to facilitate comparisons across studies and across countries [52].

All the articles reviewed utilized data generated from well-defined catchment areas managed by either the KEMRI and CDC, or the KEMRI and Wellcome Trust research collaboration; indeed a majority of the articles reviewed included KEMRI and CDC co-authors who are also authors on this paper. Additionally, all the papers that we reviewed included in their analysis data that were collected year-round and a majority of them had multiple years of data used in estimating incidence rates. This consideration is important because–other than considering that influenza circulates in Kenya year-round [7]–it minimizes the risk of overestimating the disease burden by only sampling during epidemic periods.

Six of the ten studies reviewed utilized mid-year population denominators, derived from HDSS or National census data [12], for the estimation of incidence rates. As opposed to using denominators derived from individual follow-up (person-time) in population-based surveillance systems, denominators based on mid-year population could potentially underestimate the incidence rates as they may not accurately reflect the actual population dynamics relating to births, migrations and deaths, especially if smaller populations are involved [53]. However, using either of these two denominator types to estimate incidence rates would normally yield nearly identical results for large populations. Taken in the context of the resources required to set-up and run a population-based surveillance system, denominators derived from mid-year population numbers may be useful for disease burden estimation in Kenya for the foreseeable future.

Only one study reported rates among children <6 months, and a few reported data on those aged ≥50 years [11, 12] which is in part explained by the lower health-seeking behavior among older persons in Kenya [49], and perhaps also by a diminished likelihood that older patients will report the fever required to meet the WHO SARI case definition. Understanding the disease burden, especially in the high risk groups which include pregnant women, and people with underlying medical conditions; as well as understanding the socio-economic (direct and in-direct) burden of influenza in Kenya would be helpful to public-health and influenza control programs and understanding the impact of influenza. For example a recent study conducted in Western Kenya showed a substantial burden of influenza (3-times higher) among HIV-infected adults aged ≥18 compared to their HIV-negative counterparts [13]. Another study in South Africa reported 4–8 times greater incidence of acute lower respiratory tract infection (LRTI) with influenza among HIV-infected compared to HIV-uninfected persons [40].

Our study was subject to limitations. First, we may have missed some articles as we limited our review to only published data searched the PubMed and EMBASE databases. However, we believe that the likelihood of finding additional data relevant to our study in other databases is very low. Second, most of the published data summarized in our review included data from the 2009 pandemic influenza period and may have served to overestimate the seasonal influenza disease burden. Third, the reviewed papers were limited to respiratory surveillance only. For some populations (particularly young infants) presentation may be fever without respiratory symptoms. As such, the true burden among children may have been underestimated. Fourth, the clinical threshold to hospitalize in Kenya may not be comparable to US or Europe and therefore hospitalization rates should be interpreted with caution when making these comparisons. Fifth, most of the studies presented data on incidence of influenza without presenting either the age-specific denominators or age-specific numbers of cases. Taken together with the fact that there were varied case definitions and adjustments applied, this made it difficult for us to calculate meta-analytic rates of influenza disease burden in Kenya. Lastly, while not a direct limitation of our methods, the absence of data on influenza mortality remains a gap that needs to be addressed in order to inform influenza vaccine policy.

In conclusion, our literature review provides a comprehensive summary of available data on the disease burden of influenza in Kenya over the past 8 years, and shows a substantial medically- and non-medically attended disease burden among children aged <5 years. Additional research gaps identified in the review include the lack of influenza mortality and socio-economic disease burden data. While these additional data would be very helpful to policy makers and other stakeholders to inform prevention and treatment policies, the current data in Kenya indicate an important burden of influenza in young children that might be reduced with a targeted vaccination program including children and pregnant women. However, any decision about influenza vaccination must look at its burden relative to other respiratory pathogens such as respiratory syncytial virus–when a vaccine becomes available—and even non-respiratory vaccine preventable diseases.

## Supporting Information

S1 PRISMA Checklist PRISMA 2009 Checklist. (DOC)

S1 Table Supporting tables. (DOCX)

S1 File Summarized equations for the adjustment factors. (DOCX)
