# Supplementary material for: Influenza-Associated Disease Burden in Kenya: A Systematic Review of Literature
Source: PLoS One. 2015 Sep 23;10(9):e0138708. doi: 10.1371/journal.pone.0138708 (PMC4580615; doi:10.1371/journal.pone.0138708)
Supplement: S1 Table — (DOCX) [file pone.0138708.s002.docx]

**S1 Tables. Supporting tables**

**S1 Table A.** Case definitions use for respiratory syndromes in reviewed articles

| **Authors(s)** | **Syndrome** | **Case definitions used** |
| --- | --- | --- |
| Berkley et al. (2010) | Hospitalized Severe or very severe pneumonia | Severe pneumonia was defined as cough OR difficult breathing AND lower chest wall in-drawing and no signs of very severe pneumonia.  Very severe pneumonia was defined as cough OR difficult breathing AND at least one of ( hypoxia, inability to drink or breast feed, inability to sit, or impaired consciousness at admission). |
| Onyango et al. (2012) | Hospitalized Severe or very severe pneumonia | Severe pneumonia was defined as cough OR difficulty breathing AND lower-chest-wall in-drawing.  Very severe pneumonia was defined as cough OR difficulty breathing AND one or more of (cyanosis, prostration, unconsciousness, or an oxygen saturation level <90%). |
| Ahmed et al. (2012) | Hospitalized SARI | For children > 1 week and < 2 months old, SARI was defined as an admission to the pediatric ward with any of the following: respiratory rate > 60 per minute, severe chest in-drawing, nasal flaring, grunting, fever ≥ 38°C, hypothermia < 35.5°C, or pulse oxygenation < 90%.  For children 2 months to < 5 years of age, SARI was defined as cough or difficulty breathing and any one of the following: respiratory rate > 50/min for infants 2 months to < 1 year old or > 40/min for children 1 to < 5 years old, chest in-drawing or stridor in a calm child, unable to drink or breast feed, vomiting, convulsions, lethargic or unconscious, or pulse oxygen saturation < 90%.  For older children and adults ≥ 5 years of age, SARI was defined as fever ≥ 38°C, and cough or sore throat, and shortness of breath or difficulty breathing. |
| Fuller et al. (2013) | Hospitalized SARI | For children <5 years as cough or difficulty breathing and any one of the following: IMCI^ǂ^ danger sign, tachypnea for age group, nasal flaring, grunting, oxygen saturation <90%, chest in-drawing, or stridor in a calm child. In patients aged ≥5 years, SARI was defined as any hospitalized case with cough, difficulty breathing, or chest pain during the previous 14 days. |
| Emukule et al. (2014) | Hospitalized SARI | Defined as cough or difficulty breathing or pleural chest pain within the last 14 days for persons of all ages. |
| Feikin et al. (2012) | Hospitalized ARI | Acute cough, difficulty in breathing or pleuritic chest pain. |
| Feikin et al. (2013) | In- and outpatient ALRI | For patients aged ≥5 years, ALRI was defined as cough, difficulty breathing or chest pain and either documented axillary temperature ≥38°C or oxygen saturation <90%. |
| Katz et al. (2012) | In- and outpatient ALRI | For in- and out-patient children <5 years ALRI was defined as cough OR difficulty breathing, AND at least one of (maternal report of IMCI^ǂ^ danger sign, lower-chest wall in-drawing, stridor, oxygen saturation <90%).  For in- and out-patients aged ≥5 years, ALRI was defined as cough OR difficulty breathing OR chest pain, AND a documented axillary temperature of ≥38.0°C OR and oxygen saturation level of ≤90%. |
| Breiman et al. (2015) | Outpatient SARI | For in- and out-patient children <5 years SARI was defined as cough OR difficulty breathing, AND at least one of (unable to drink/breastfeed, vomits everything, convulsions, lethargic or unconscious, stridor when calm, and lower chest wall in-drawing, as well as an additional criterion of oxygen saturation <90%). |
| Katz et al. (2012) | In- and outpatient ILI | Defined as axillary temperature ≥38°C AND cough or sore throat in an outpatient within the past 14 days for persons of all ages. |
| Emukule et al. (2014) | Outpatient ILI | Defined as axillary temperature ≥ 38°C and cough or sore throat for persons of all ages. |
| Feikin et al. (2012) | In- and outpatient ARI | Cough or difficulty breathing or chest pain and documented axillary temperature ≥38°C or oxygen saturation <90% or hospitalization. |
| Fuller et al. (2013) | Non-hospitalized SARI | Those with pneumonia who did not seek care in health utilization survey (HUS). In this HUS, pneumonia was defined as: cough or difficulty breathing for more than two days or a diagnosis of ‘pneumonia’ by a healthcare worker. |
| Emukule et al. (2014) | Non-hospitalized SARI | Those with pneumonia who did not seek care in health utilization survey (HUS). In this HUS, pneumonia was defined as: cough and difficulty in breathing for more than two days (excluding the past 14 days) within the preceding 12 months or a diagnosis of ‘pneumonia’ by a healthcare worker. |
| Emukule et al. (2014) | Non-medically attended ILI | Those with acute respiratory illness (ARI) who did not seek care in a HUS. In this HUS, ARI was defined as: cough and difficulty in breathing within the last 14 days. |

^ǂ^Inability to drink or breastfeed, vomiting everything, convulsions, lethargy or unconscious.

**S1 Table B.** Proportion testing positive for influenza A and/or B among hospitalized patients and in- and outpatients seen with different respiratory syndromes in Kenya

| Author(s) | Study period | Syndrome type | Study site | Age group | Influenza positive n/N(%) |
| --- | --- | --- | --- | --- | --- |
| Berkley et al. (2010) | Jan to Dec, 2007 | Hospitalized severe or very severe pneumonia | Kilifi | <5 yrs | 44/779(5.8)^a^ |
| Onyango et al. (2012) | Jan, 2007 to Dec, 2010 | Hospitalized severe or very severe pneumonia. | Kilifi | <5 yrs | 99/2,002(4.9) |
| Ahmed et al. (2012) | Sep, 2007 to Aug, 2010 | Hospitalized SARI | Kakuma and Dadaab | <5 yrs | 410/4,449(9.2)^a^ |
| Emukule et al. (2014) | Aug, 2009 to Jul, 2012 | Hospitalized SARI | Siaya | All ages | 348/5,507(7.9) |
| Feikin et al. (2012) | Mar, 2007 to Feb 2010 | Hospitalized ARI | Bondo | <5 yrs | 83/1,213(6.8) |
|  |  |  |  | ≥5 yrs | 121/866(14.0) |
|  |  |  |  | All ages | 204/2079(9.8) |
| Katz et al. (2012) | Mar, 2007 to Feb 2010 | In- and outpatient ALRI | Kibera | All ages | 319/1197(26.7) |
|  |  |  | Lwak | All ages | 359/1,641(21.9) |
| Feikin et al. (2013) | Mar, 2007 to Feb 2010 | In- and outpatient SARI | Lwak | <5 yrs | 27/408(6.6)^a^ |
| Breiman et al. (2015) | Mar, 2007 to Feb 2011 | Outpatient SARI | Kibera | <5 yrs | 112/818(13.7) |
| Feikin et al. (2012) | Mar, 2007 to Feb 2010 | In- and outpatient ARI | Lwak | ≥5 yrs | 249/1216(20.5)^a^ |
| Emukule et al. (2014) | Aug, 2009 to Jul, 2012 | Outpatient ILI | Ting'wang'i | All ages | 206/1,632(13.7) |

Abbreviations: SARI=Severe acute respiratory illness; ALRI=Acute lower respiratory illness; ILI=influenza-like illness; ARI=Acute respiratory illness

**S1 Table C.** Average annual incidence rates of influenza-associated hospitalizations for different respiratory syndromes (per 1,000 persons or person-years) in Kenya

| **Author(s)** | **Study period** | **Syndrome type** | **Adjustment used** | **Study site** | **Age group** | **Incidence***  **(95% CI)** |
| --- | --- | --- | --- | --- | --- | --- |
| Berkley et al. (2010) | Jan to Dec, 2007 | Hospitalized Severe or very severe pneumonia | None stated. | Kilifi^¢^ | < 1 yr | 2.40^a^ |
|  |  |  |  |  | 1-<2 yrs | 1.00^a^ |
|  |  |  |  |  | 2-4 yrs | 0.40^a^ |
|  |  |  |  |  | <5 yrs | 0.80^a^ |
| Onyango et al. (2012) | Jan, 2007 to Dec, 2010 | Hospitalized severe or very severe pneumonia. | None stated. | Kilifi | <1 yrs | 1.5(1.2-2.0) |
|  |  |  |  |  | <5 yrs | 0.6(0.5-0.7) |
| Ahmed et al. (2012) | Sep, 2007 to Aug, 2010 | Hospitalized SARI | None stated. | Kakuma refugee camp | < 1 yr | 12.3(7.7-19.5)^a^ |
|  |  |  |  |  | 1-4 yrs | 4.2(2.9-6.1)^a^ |
|  |  |  |  |  | < 5 yrs | 5.6(4.2-7.5)^a^ |
|  |  |  |  | Dadaab refugee camp | < 1 yr | 10.3(6.8-15.6)^a^ |
|  |  |  |  |  | 1-4 yrs | 2.9(2.1-4.2)^a^ |
|  |  |  |  |  | < 5 yrs | 4.2(3.2-5.5)^a^ |
| Fuller et al. (2013) | Aug, 2009 to Jul, 2010 | Hospitalized SARI | Rates adjusted for risk factor and healthcare-seeking for SARI (See the online appendix of the published paper). | Siaya | <5 yrs | 3.9(3.1-4.7) |
|  |  |  |  |  | ≥5 yrs | 0.3(0.2-0.4) |
|  |  |  |  | Kenya | <5 yrs | 4.7(3.5-6.2) |
|  |  |  |  |  | ≥5 yrs | 0.2(0.2-0.3) |
|  |  |  |  |  | All ages | 1.1(0.9-1.6) |
|  | Aug, 2010 to Jul, 2011 | Hospitalized SARI | Rates adjusted for risk factor and healthcare-seeking for SARI (See the online appendix of the published paper). | Siaya | <5 yrs | 3.0(2.2-3.7) |
|  |  |  |  |  | ≥5 yrs | 0.4(0.3-0.5) |
|  |  |  |  | Kenya | <5 yrs | 3.0(2.3-3.9) |
|  |  |  |  |  | ≥5 yrs | 0.2(0.2-0.4) |
|  |  |  |  |  | All ages | 0.7(0.5-0.9) |
| Emukule et al. (2014) | Aug, 2009 to Jul, 2012 | Hospitalized SARI | Rates adjusted for those hospitalized with SARI who did not have swabs tested for influenza virus. | Siaya | <6 mos | 5.7(2.4-13.8) |
|  |  |  |  |  | 6-11 mos | 4.7(1.8-11.9) |
|  |  |  |  |  | 12-23 mos | 4.5(2.3-8.6) |
|  |  |  |  |  | 2-4 yrs | 1.4(0.7-2.8) |
|  |  |  |  |  | <5 yrs | 2.7(1.8-3.9) |
|  |  |  |  |  | ≥5 yrs | 0.3(0.2-0.4) |
|  |  |  |  |  | All ages | 0.7(0.5-0.9) |
| Feikin et al. (2012) | Mar, 2007 to Feb 2010 | Hospitalized ARI | Rates adjusted for those hospitalized with ARI who did not have swabs tested for influenza. | Bondo | <1 yr | 1.4(0.9-1.8) |
|  |  |  |  |  | <5 yrs | 1.4(1.2-1.7) |
|  |  |  |  |  | All ages | 0.6(0.5-0.6) |

Abbreviations: SARI=Severe acute respiratory illness; ARI=Acute respiratory illness.

*Incidence reported per 1,000 persons or person-years; ^£^SARI in the community was defined as: cough or difficulty breathing AND one of (chest wall in-drawing, vomiting everything, lethargic, convulsions, or inability to drink or breast feed); ^a^Influenza A; ^¢^95% CI not provided.

**S1 Table D.** Average annual incidence rates of medically-attended influenza A and/or B (hospitalized and outpatient) per 1,000 persons or person-years in Kenya

| **Author(s)** | **Study period** | **Syndrome type** | **Adjustment used** | **Study site** | **Age group** | **Incidence***  **(95% CI)** |
| --- | --- | --- | --- | --- | --- | --- |
| Katz et al. (2012) | Mar, 2007 to Feb 2010 | In- and outpatient ALRI | Rates adjusted for those with ALRI at the study clinic who were not tested for influenza. | Kibera | < 1 yr | 32.8(21.4-50.2) |
|  |  |  |  |  | 1-<2 yrs | 26.2(18.3-37.5) |
|  |  |  |  |  | 2-4 yrs | 17.1(12.6-23.1) |
|  |  |  |  |  | <5 yrs | 22.0(17.7-26.6) |
|  |  |  |  |  | ≥5 yrs | 12.0(10.3-13.3) |
|  |  |  |  |  | All ages | 13.7(12.2-15.2) |
|  |  |  |  | Lwak | < 1 yr | 42.1(22.7-78.3) |
|  |  |  |  |  | 1-<2 yrs | 43.9(26.0-74.1) |
|  |  |  |  |  | 2-4 yrs | 40.1(28.3-56.7) |
|  |  |  |  |  | <5 yrs | 40.5(31.2-52.6) |
|  |  |  |  |  | ≥5 yrs | 15.8(14.1-17.7) |
|  |  |  |  |  | All ages | 23.0(20.8-25.5) |
| Feikin et al. (2013) | Mar, 2007 to Feb 2010 | In- and outpatient SARI | Rates were adjusted for healthcare seeking by extrapolating from those with ARI^ǂ^ at household visit who sought care at a clinic besides the study clinic and for the pathogen-attributable fraction (PAF^¥^). | Lwak | <5 yrs | 58.0(38.0-78.0) |
| Breiman et al. (2015) | Mar, 2007 to Jul, 2011 | Outpatient SARI | Adjusted for healthcare seeking for SARI at the study clinic and for the pathogen-attributable fraction (PAF^¥^). | Kibera | <5 yrs | 13.0(6.0-20.0) |
| Feikin et al. (2012) | Mar, 2007 to Feb 2010 | In- and outpatient ARI | Rates were adjusted for healthcare seeking by extrapolating from those with ARI^ǂ^ at household visit who sought care at a clinic besides the study clinic and for the pathogen-attributable fraction (PAF^¥^). | Lwak | ≥5 yrs | 26.0(22.8-29.2)^a^ |
| Emukule et al. (2014) | Aug, 2009 to Jul, 2012 | Outpatient ILI | Rates adjusted for those with ILI at the outpatient clinic who did not have swabs tested for influenza. | Ting'wang'i | <6 mos | 16.2(3.5-73.8) |
|  |  |  |  |  | 6-11 mos | 37.7(14.7-96.7) |
|  |  |  |  |  | 12-23 mos | 31.8(15.6-64.4) |
|  |  |  |  |  | 2-4 yrs | 17.2(10.3-28.9) |
|  |  |  |  |  | <5 yrs | 21.8(15.1-31.6) |
|  |  |  |  |  | ≥5 yrs | 4.3(2.8-6.4) |
|  |  |  |  |  | All ages | 7.2(5.5-9.4) |

Abbreviations: SARI=Severe acute respiratory illness; ALRI=Acute lower respiratory illness; ILI=influenza-like illness; ARI=Acute respiratory illness

*Incidence reported per 1,000 persons or person-years; ^ǂ^ARI in home was defined as cough, difficulty breathing or chest pain and reported fever; ^¥^Adjusted rates downward for asymptomatic detection of influenza in controls; ^a^Influenza A.

**S1 Table E.** Non-medically attended average annual incidence rates of Influenza reported for different respiratory syndromes (per 1,000 persons or person-years) in Kenya

| **Author(s)** | **Study period** | **Syndrome type** | **Adjustment used** | **Study site** | **Age group** | **Incidence* (95% CI)** |
| --- | --- | --- | --- | --- | --- | --- |
| Fuller et al. (2013) | Aug, 2009 to Jul, 2010 | Non-medically attended SARI | Percent of pneumonia case hospitalized from health utilization survey (HUS). | Siaya | <5 yrs | 4.2(2.7-7.3) |
|  |  |  |  |  | ≥5 yrs | 0.6(0.3-1.2) |
|  |  |  |  | Kenya | <5 yrs | 5.1(3.5-8.1) |
|  |  |  |  |  | ≥5 yrs | 0.4(0.2-0.8) |
|  |  |  |  |  | All ages | 1.4(0.9-2.4) |
|  | Aug, 2010 to Jul, 2011 | Non-medically attended SARI | Percent of pneumonia case hospitalized from HUS. | Siaya | <5 yrs | 3.2(2.1-5.5) |
|  |  |  |  |  | ≥5 yrs | 0.8(0.5-1.6) |
|  |  |  |  | Kenya | <5 yrs | 3.3(2.4-5.2) |
|  |  |  |  |  | ≥5 yrs | 0.5(0.3-0.9) |
|  |  |  |  |  | All ages | 0.9(0.6-1.6) |
| Emukule et al. (2014) | Aug, 2009 to Jul, 2012 | Non-medically attended SARI | Adjusted for persons with pneumonia who did not seek care, using the results of a 2005 HUS. | Siaya | <6 mos | 6.2(2.9-13.2) |
|  |  |  |  |  | 6-11 mos | 5.0(2.2-11.4) |
|  |  |  |  |  | 12-23 mos | 4.8(2.7-8.6) |
|  |  |  |  |  | 2-4 yrs | 1.6(0.9-2.7) |
|  |  |  |  |  | <5 yrs | 2.9(2.1-4.0) |
|  |  |  |  |  | ≥5 yrs | 0.5(0.4-0.7) |
|  |  |  |  |  | All ages | 1.2(0.9-1.4) |
| Emukule et al. (2014) | Aug, 2009 to Jul, 2012 | Non-medically attended ILI | Adjusted for persons with ARI who did not seek care, using the results of a 2005 HUS. | Ting'wang'i | <6 mos | 22.3(15.0-33.2) |
|  |  |  |  |  | 6-11 mos | 52.1(40.4-67.1) |
|  |  |  |  |  | 12-23 mos | 43.8(36.3-53.0) |
|  |  |  |  |  | 2-4 yrs | 23.8(20.7-27.4) |
|  |  |  |  |  | <5 yrs | 30.1(27.3-33.3) |
|  |  |  |  |  | ≥5 yrs | 5.4(4.9-6.0) |
|  |  |  |  |  | All ages | 9.1(8.5-9.8) |

*Incidence reported per 1,000 persons or person-year.
