# Supplementary material for: Influenza-Associated Disease Burden in Kenya: A Systematic Review of Literature
Source: PLoS One. 2015 Sep 23;10(9):e0138708. doi: 10.1371/journal.pone.0138708 (PMC4580615; doi:10.1371/journal.pone.0138708)
Supplement: S1 File — (DOCX) [file pone.0138708.s003.docx]

**S1 File**. **Summarized equations for the adjustment factors accounting for persons who were not tested for influenza, those who did not seek healthcare, and for asymptomatic detection of influenza**

**Equation 1.** Incidence rate of influenza-associated hospitalization/outpatient visit

$$IR=\frac{{Flu}_{cases}}{PoY}$$

Where:

$IR$ = Unadjusted incidence rate of influenza-associated hospitalizations/outpatient visits

${Flu}_{cases}$ = Total number of cases who tested positive for influenza

*PoY*= Person-time of surveillance in years/Mid-year population

**Equation 2a.** Adjustment for those who met the specific case definition but were not tested for influenza

$$AdjIR=\left( IR\times\frac{1}{P_{flutest}} \right)$$

Where:

$AdjIR$ = Incidence adjusted for those who were not tested for influenza

$IR$ = Unadjusted (crude) incidence rate of influenza-associated hospitalizations/outpatient visits

$P_{flutest}$ = Proportion of hospitalized patients who met the specific case definition and were tested for influenza

**Equation 2b.** Adjustment for those who did not seek healthcare

$$AdjIR=\left( IR\times\frac{1}{P_{case}} \right)$$

Where:

$AdjIR$ = Incidence adjusted for those who were not tested for influenza

$IR$ = Unadjusted (crude) incidence rate of influenza-associated hospitalizations/outpatient visits

$P_{case}$= Proportion of cases in the community who met the specific case definition and sought healthcare at the study hospital/clinic

**Equation 2c.** Adjustment for asymptomatic detection of influenza

$$AdjIR=\left( IR\times PAF \right)$$

Where:

$AdjIR$ = Incidence adjusted for those who were not tested for influenza

$IR$ = Unadjusted (crude) incidence rate of influenza-associated hospitalizations/outpatient visits

$PAF$= Pathogen-attributable fraction defined as (OR-1)/OR

$OR$= The odds ratio of detection of influenza among cases compared to asymptomatic controls from a case-control analysis

**Note**: Refer to the individual published papers for further details on specific adjustments used.
